# Supplementary material for: Biological oxygen demand optode analysis of coral reef-associated microbial communities exposed to algal exudates
Source: PeerJ. 2013 Jul 16;1:e107. doi: 10.7717/peerj.107 (PMC3719127; doi:10.7717/peerj.107)
Supplement: Figure S1 — Originally written by Keoeeit and upgraded by Alex Hewitt to incorporate battery saving options. Written in Basic. [file peerj-01-107-s001.docx]

rem Author - Keoeeit

rem Upgraded by Mika Tanninen

rem updated by Alex Hewitt

rem updated by Allison Gregg

@title Ultra Intervalometer

@param a Delay 1st Shot (Mins)

@default a 0

@param b Delay 1st Shot (Secs)

@default b 0

@param c Number of Shots (0 inf)

@default c 0

@param d Interval (Minutes)

@default d 1

@param e Interval (Seconds)

@default e 0

@param f Interval (10th Seconds)

@default f 0

n=0

z=0

click "display"

t=(d*600+e*10+f)*100

if c<1 then let c=0

if t<100 then let t=100

g=(a*60)+b

if g<=0 then goto "interval"

for m=1 to g

print "Intvl Begins:", (g-m)/60; "min", (g-m)%60; "sec"

sleep 930

next m

:interval

n=n+1

if c=0 then print "Shot", n else print "Shot", n, "of", c

press "shoot_half"

sleep 2500

set_focus z

press "shoot_full"

release "shoot_full"

release "shoot_half"

if n=c then end

sleep t

goto "interval"
