# Supplementary figures and images for: Biological oxygen demand optode analysis of coral reef-associated microbial communities exposed to algal exudates
Source: PeerJ. 2013 Jul 16;1:e107. doi: 10.7717/peerj.107 (PMC3719127; doi:10.7717/peerj.107)

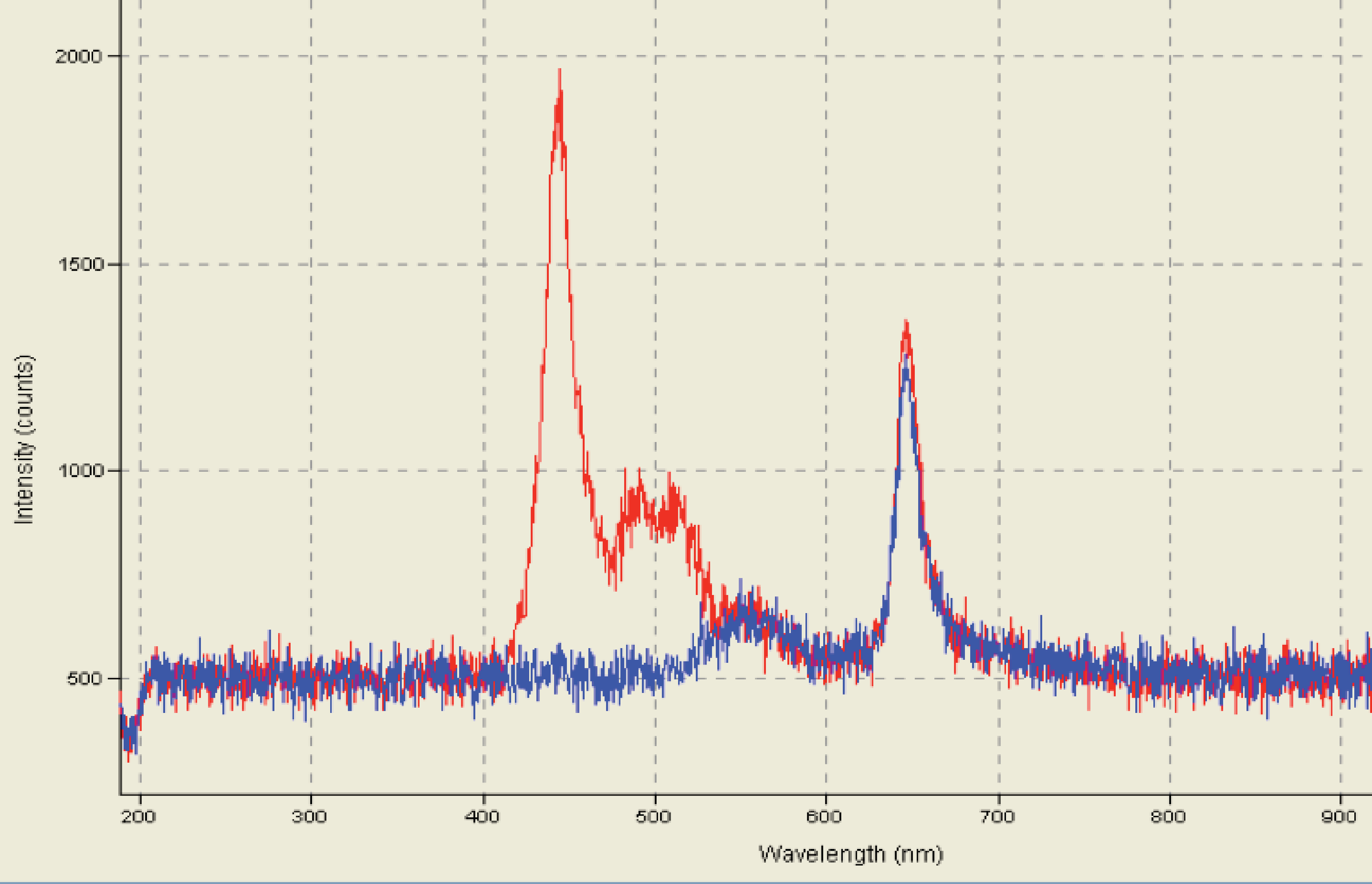

Supplement: Figure S2 — Optode exposed to ambient air conditions. Blue and red spectrum corresponds to oxygen optode signal with and without 530 nm long pass filter, respectively. [file peerj-01-107-s002.png]
